# Modulation of Macrophage Function by *Lactobacillus*-Conditioned Medium

**DOI:** 10.3389/fcell.2020.00723

**Published:** 2020-07-30

**Authors:** Yashaswini Seenappanahalli Nanjundaiah, David A. Wright, Anwar R. Baydoun, Zahangir Khaled, Zulfiqur Ali, Paul Dean, Mosharraf H. Sarker

**Affiliations:** ^1^School of Health and Life Sciences, Teesside University, Middlesbrough, United Kingdom; ^2^Faculty of Health and Life Sciences, De Montfort University, Leicester, United Kingdom; ^3^Pediatric Gastroenterology, College of Medicine at Peoria, University of Illinois, Peoria, IL, United States

**Keywords:** *Lactobacillus rhamnosus* GG, macrophage, free radicals, phagocytosis, nitric oxide

## Abstract

Probiotics are used as microbial food supplements for health and well-being. They are thought to have immunomodulatory effects although their exact physiological mechanism of action is not clear. This study investigated the influence of probiotic *Lactobacillus rhamnosus* GG conditioned media (LGG-CM) on macrophage phagocytosis of non-pathogenic *Escherichia coli* HfrC. The gentamicin protection assay was used to study the bacterial killing phases of phagocytosis. Macrophages co-incubated with *E. coli* for an hour allowed them to ingest bacteria and then the rate of *E. coli* killing was monitored for up to 300 min to determine the killing or digestion of the bacteria by recovering them from the macrophage lysate. We found that the LGG-CM significantly increased the bacterial killing by approximately 6-fold when compared with that of controls. By contrast, this killing process was found to be associated with enhanced free radical production via the activation of NADPH oxidase, stimulated by the LGG conditioned medium. We also found that the conditioned medium had small effect on nitric oxide (NO) generation, albeit to a lesser extent. This work suggests that LGG-CM may play an important role in suppressing the total microbial load within the macrophages and hence, the extent to which pro-inflammatory molecules such as free radicals and NO are generated. The modulation of inflammation-promoting signals by LGG-CM may be beneficial as it modulates bacterial killing, and thereby prevents any collateral damage to host.

## Introduction

There is considerable interest in the role of probiotic bacteria in the modulation of human health and immune function (Yan and Polk, 2012). Species of lactic acid bacteria have been suggested to promote host innate immune function by influencing activity of phagocytic cells and modulating pathogen-induced inflammatory responses (Donnet-Hughes et al., 1999; Gill et al., 2001; Kotzamandis et al., 2010; Marranzino et al., 2012). Daily ingestion of probiotic supplements has also been shown to enhance innate immune functions by significantly increasing the number of phagocytic cells and IgA+ antibody levels in the blood (Berman et al., 2006; Galdeano et al., 2011).

Regulating phagocytic cell activity by probiotics may enhance the ability to engulf and kill harmful bacteria which is an important and highly conserved innate immune mechanism carried out by phagocytes such as macrophages and neutrophils. Phagocytosis follows a complex series of steps starting with the recognition and ingestion of the microbe, and formation of a phagosome where the microbe is eventually killed. Immunological recognition of microorganisms by phagocytes is followed by a series of biochemical events, producing a plethora of microbicidal agents including reactive oxygen species (ROS), nitric oxide (NO), and other reactive nitrogen species (RNS) and lytic enzymes which contribute to bacterial death (Flannagan et al., 2009).

While the health benefits of probiotics are becoming more evident, little is known of their underlying mechanisms of action. Moreover, current literature on their role in regulating phagocytosis have mostly looked at either the uptake of total number of particles or microbes, or the upregulation of oxidative activities of the cells when exposed to pathogen. There is limited literature on their role in the digestion or killing phases of phagocytosis (Yan and Polk, 2002; Vincenti, 2010). The aim of this study therefore was to investigate the effect of *Lactobacillus* on the killing phases of phagocytosis of *Escherichia coli* by mouse macrophages. The probiotic bacterium *Lactobacillus rhamnosus GG* (LGG), an extensively studied and widely used probiotic strain of human origin, was investigated as it has been shown to enhance phagocytic activity (Pelto et al., 1998) and stimulate antibody production (Kaila et al., 1992). Clinically, *Lactobacillus* strains of probiotics have also been reported to reduce diarrheal illness caused by intestinal pathogens (Nixon et al., 2012). Additionally, there is evidence that secretory components of *Lactobacillus* may also exert a beneficial effect on host immune activities (Rudin and Lundell, 2012; Yoda et al., 2012; Villena and Kitazawa, 2014). We have therefore investigated conditioned medium obtained from cultures of LGG and examined the digestion and degree of killing of *E. coli* induced by free radicals during phagocytosis.

## Materials and Methods

### Macrophage and Bacterial Culture

The murine macrophage cell line J774A.1 was acquired from the American Type Culture Collection (ATCC) and used in all macrophage assays. Cells were cultured in Dulbecco’s modified Eagle medium (DMEM; Gibco) with high glucose, supplemented with 10% FBS and 100 μg/mL penicillin/streptomycin (Sigma) and incubated in a humidified incubator at 37°C and 5% CO_2_. Macrophages were seeded into a 24-well plate at 5 × 10^5^ cells/well. LGG was grown from commercially available Culturelle^®^ tablets in de Man, Rogosa and Sharpe (MRS) broth. The Gram-negative bacterium *E. coli* strain HfrC, obtained from the National Collection of Industrial, Food and Marine Bacteria (NCIMB, United Kingdom), was used in all gentamycin protection assays following overnight growth in Luria-Bertani (LB) broth (Oxoid).

### Production of *Lactobacillus rhamnosus* GG Conditioned Media

The *Lactobacillus rhamnosus* GG Conditioned Media (LGG-CM) was prepared as described previously (Nanjundaiah et al., 2016). In brief, under sterile conditions, a capsule of LGG Culturelle^®^ was inoculated into 100 ml of MRS broth and incubated for 24 h at 37°C with oscillation at 150 rpm to early stationary phase. The LGG culture was then centrifuged for 10 min at 10,000 × *g* and the supernatant discarded. The pellet was resuspended in 50 ml DMEM and incubated for a further 24 h at 37°C with oscillation at 150 rpm. Cell-free supernatant was obtained by centrifugation for 10 min at 10,000 rpm and filtered through 0.22 μm filters. The supernatant was then aliquoted into small microfuge tubes and stored at −20°C for further use. For each replica experiment, different batches of LGG-CM were prepared.

### Macrophage Viability Assay

Macrophage viability was monitored by the mitochondrial-dependant 3-(4,5-dimethyl-2-thiazyl)-2,5-diphenyl-2H-tetrazo- lium bromide (MTT) reduction assay. This test is based on the cleavage of the tetrazolium salt MTT to yield a blue formazan dye in viable cells. J774 macrophages (1 × 10^5^ cells/mL) were seeded 4 h prior to the assay in 96-well plates. LGG-CM was added to the cells at dilutions of 10, 75, and 100% with or without LPS (20 μg/ml) and incubated for 6 or 24 h. MTT solution (5 mg/mL; Sigma) was prepared and added to each well according to manufacturer’s instructions. The A_550_ was measured using a ELx 800 microplate reader (Bio-Tek) and % cytotoxicity was determined relative to control (untreated) cells. Lipopolysaccharide (LPS) from *E. coli* O111:B4 (purchased from Sigma) was used as a positive control for evaluation of macrophage activation.

### Experiments Investigating Antimicrobial Properties of LGG-CM

To determine whether the LGG-conditioned medium demonstrated antibacterial properties within the specified time frame of our experiments, *E. coli* HfrC was grown overnight in LB broth at 37°C with shaking. A 1 ml sample of *E. coli* culture was centrifuged and the pellet was carefully washed with PBS followed by DMEM and resuspended in 1 ml of DMEM devoid of penicillin/streptomycin. This was diluted 1:50 in 1 ml of varying concentrations of LGG-CM (5, 10, and 75, and 100% LGG-CM, pH 7.4 ± 0.2) for 6 or 24 h, after which, the bacteria was serially diluted and plated onto nutrient agar plates and colony forming (CFU) were counted.

### Monitoring Macrophage Killing of Intracellular *E. coli*

Macrophages were grown in 24-well plates at 5 × 10^5^ cells per well and incubated with *E. coli* in the presence of DMEM alone (control) or with LPS (20 μg/mL), LGG conditioned medium (LGG-CM; 10, 75, and 100%) or a combination of both. An *E. coli* multiplicity of infection (MOI) of 50:1 was used for all experiments. Following an uptake period of 60 min, gentamicin (200 μg/mL) was added for 15 min to kill all extracellular bacteria. Cells were washed with sterile PBS and incubated in DMEM containing a low inhibitory dose of gentamicin (20 μg ml^–1^). Cells were incubated for an indicated time period after which the macrophages were washed, lysed in 0.1% (v/v) triton-X 100 and the released bacteria were resuspended and serially diluted, before being enumerated by CFU counts on nutrient agar plates. Time course experiments were performed every 40 min and data were fitted to killing curve using the equation a = a^0^e^–*kt*^.

For inhibitor studies, macrophages were plated at a cell density of 5 × 10^5^ cells/mL and pre-treated with different inhibitors including 100 μM L-NMMA (inhibitor of nitric oxide synthase), 100 μM apocynin (NADPH oxidase inhibitor) or a combination of 100 μM L-NMMA plus apocynin for 1 h. Cell permeable forms of superoxide dismutase and catalase fused to polyethylene glycol (Sigma) were used to scavenge free radical during the assay. After pre-treatment the inhibitors were removed and cells were washed with PBS prior to the gentamycin protection assay (described above).

### Staining and Visualizing Live and Dead Intracellular Bacteria

Macrophages were cultured as described above using glass bottom culture dishes for imaging. An overnight culture of *E. coli* was washed and labeled with 0.01% (w/v) acridine orange for 45 s. The stained cells were then washed 3 times with PBS and added (50:1) to the macrophages and incubated for 60 min. After the coincubation period, cells were washed once with prewarmed PBS and extracellular bacteria were killed by gentamicin treatment. For monitoring bacterial digestion, images were taken at 15–300 min after a coincubation period of 60 min after which 0.05% (w/v) crystal violet dissolved in PBS was added to the cells to quench any extracellular fluorescence. Images were taken using Open Lab software and analyzed using Image Hopper 2 (Samsara research, United Kingdom). Crystal violet does not penetrate intact macrophage membranes and therefore does not quench the fluorescence of intracellular bacteria. The viable ingested bacteria appear green and dead bacteria appear red (Takao et al., 1996).

### ROS Detection Using a Microplate Reader

Accumulation of intracellular ROS produced by J774 murine macrophages in 96 well tissue culture plates was measured using 2,7-dichlorofluorescein diacetate (H_2_DCFDA) and a microplate reader (Biotek). After removing the culture medium, the cells were washed once with pre-warmed PBS. Macrophages were then loaded with fresh medium containing 5 μM H_2_DCFDA for 45 min at 37°C in 5% CO_2_ in a humidified cell culture incubator. The dye solution was then removed and cells again carefully washed twice with pre-warmed PBS. The fluorescence measurements were taken every 2 min approximately for 280 min to monitor ROS production. The fluorescence was measured at 485 nm excitation and 528 nm emissions.

### ROS Detection Using Fluorescence Microscopy

Fluorescence imaging of intracellular ROS in macrophages was detected using H_2_DCFDA with a Nikon Eclipse TE-2000U fluorescence microscope. Macrophages were scraped, counted and 1 ml of macrophage culture added to small Petri dishes containing glass coverslips and incubated overnight in a humidified tissue culture incubator at 37°C and 5% carbon dioxide. On the day of the experiment, the media was replaced with media containing 5 μM H_2_DCFDA. The cells were then left in the cell culture incubator for 45 min, washed three times with phosphate buffered saline and fresh prewarmed phenol red free DMEM was added. The cover slip was removed and attached to the bottom of the imaging chamber (macrophage affixed side facing up) before the medium was added onto the chamber. The chamber was then placed under an inverted microscope where a bright field filter was used to focus the cells prior to the fluorescence imaging using Improvision Open lab software. A single channel heater controller (model TC-324B, Harvard Apparatus, United Kingdom) was used to control the temperature at 37°C. Fluorescence images of ROS were captured in control and LPS and LGG treated cells at various time periods. Images were captured at excitation and emission wavelength of 495 nm and 520 nm. The images were saved as tiff files a*n*d finally analyzed using Image Hopper2 software (Samsara Research, United Kingdom).

### Western Blot Analysis Expression

Lysates for Western blot analysis were prepared from cells as follows. Total cell protein was determined using bicinchoninic acid (BCA) reagents following the manufacturer’s instructions. Standard SDS-PAGE was performed and proteins were then transferred onto a polyvinylidene difluoride (PVDF) membrane and detected with either polyclonal anti-gp91^*phox*^ (Santa Cruz, SC130543) or p47^*phox*^ (Santa cruz, SC17845) antibodies. β-actin was used as a loading control using an anti-β-actin antibody. Goat-anti mouse IgG (Santa Cruz, SC-2005) antibody was used as a secondary antibody conjugated with horseradish peroxidase. Bands were visualized by enhanced chemiluminescence detection using Clarity reagent (Biorad).

### Nitrite Determination by Greiss Assay

Nitrite quantification was carried out using the Griess assay as described previously (Sun et al., 2003). Briefly, 100 μl of sample (cell culture medium) was incubated for 15 min in the dark at room temperature with 100 μl Griess reagent [1:1 ratio of 1% sulfanilamide in 10% orthophosphoric acid (reagent 1) and 0.2% napthylethylenediamine in dH_2_O (reagent 2)]. Absorbance was read at 540 nm on a fluorescent plate reader.

### Statistical Analysis

All experiments were performed a minimum of three times, with three replicates in each. One-way analysis of variance (ANOVA) and *post hoc* Tukey’s test was performed on datasets with critical values set at *p* < 0.05 with GraphPad Prism software (GraphPad Software, Inc., San Diego, CA, United States). To determine the rate of exponential decrease for bacterial killing, data were fitted to a killing curve using the equation a = a^0^e^–*kt*^.

## Results

### Effect of *Lactobacillus rhamnosus* Conditioned Medium on Macrophage Viability

**FIGURE 1:**
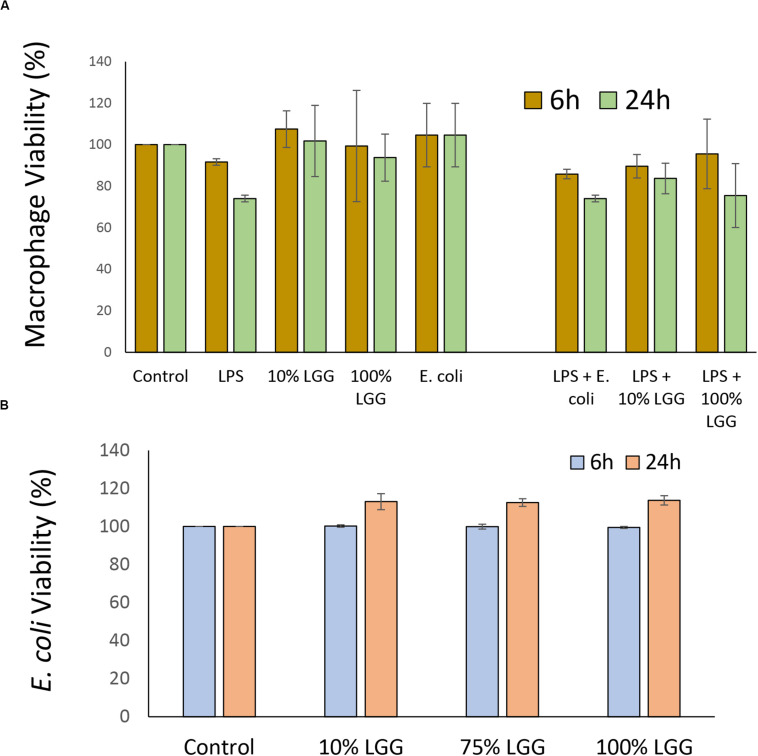
Effect of LGG conditioned medium on macrophage and *E. coli* viability. **(A)** Macrophages were grown in 24 well plates and exposed to LPS, LGG conditioned medium (LGG-CM), or *E. coli* cells for 6 h (olive), or 24 h (light green). Cell viability was assessed by MTT reduction. LPS concentration was 20 μg/ml; LGG conditioned medium was 10% or 100%. Control was no additions. **(B)** Viability of *E. coli* HfrC after incubation with LGG supernatant for 6 h (light blue) and 24 h (orange). Recovered colony forming units (CFU) were determined. Control cells had no treatment and CFU numbers were set 100%. All data shows means ± SD; experiments were performed in triplicate and repeated 3 times. One-way ANOVA showed no significant decrease in viability between any of the different conditions.

The release of immuno-modulatory compounds by the probiotic bacterium LGG was tested by growing the bacterium to a specific optical density in serum-free DMEM and testing the properties the LGG DMEM conditioned medium (LGG-CM). However, it was first necessary to test whether the LGG-CM had any biocidal activities on J774 macrophages which was determined by the MTT assay at 6 h and 24 h following post-incubation. LGG-CM had no significant effect on macrophage viability, even when used undiluted (100%) suggesting any released LGG components or LPS were not toxic to the macrophages (Figure 1A). Incubation of macrophages with LGG-CM, LPS, *E. coli* whole cells alone or a combination of these, showed little effect on macrophage viability (Figure 1A; *p* > 0.05) within the time frame. Therefore, under the experimental conditions used throughout this study, macrophage viability did not appear to be adversely affected.

As the experiments in this study involved determining *E. coli* viability, the direct effects of LGG-CM on *E. coli* survival were also examined. *E. coli* cultures grown overnight were resuspended in LGG-CM and cultured for a further 6 h and 24 h. In all cases, there was no significant adverse effects on *E. coli* growth (*p* > 0.05) at any of the LGG-CM concentrations tested suggesting that LGG-CM has no direct antibacterial effect on *E. coli* (Figure 1B).

### Macrophage Killing of *E. coli* Is Enhanced by *Lactobacillus rhamnosus* Conditioned Medium

**FIGURE 2:**
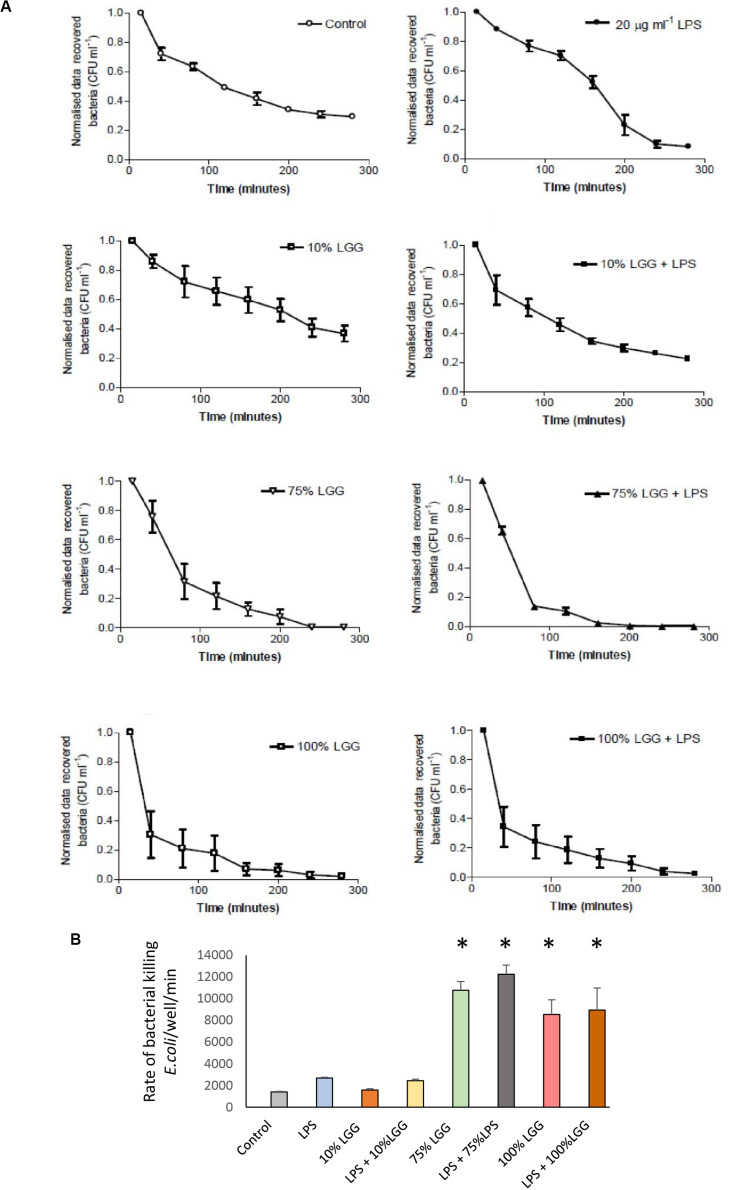
Recovered intracellular *E. coli* bacteria from J774 macrophages during bacterial killing phase. **(A)** The graphs represent the number of bacterial colonies (CFU) recovered following gentamicin protection and were normalized by converting the starting CFU/mL to 1.0 in each condition. Data shows mean ± SD; all experiments were performed in duplicate and repeated at least three times. **(B)** The rate of bacterial killing, determined as described in the materials and methods, presented in column graph format. Significant differences (*p* < 0.05) are shown with the asterisk.

The effect of the LGG-CM on the rate of bacteria killing was monitored in macrophages, with intracellular *E. coli* recovered from the macrophages every 40 min. To ensure that only intracellular bacteria were assessed, all extracellular bacteria were killed with gentamicin at 200 μg/ml. As expected, the untreated controls showed a time-dependent reduction in bacterial viability over time (1400 ± 59 bacteria killed/min; Figure 2). While 10% LGG-CM had no significant effect of the rate of bacterial killing (Figure 2; *p* > 0.05), the kill rate dramatically increased almost 10-fold following incubation in 75% or 100% LGG-CM, reaching over 12000 ± 800 bacteria killed/min). Therefore, the data strongly suggest that cell-free compounds released into the supernatant during LGG growth stimulate macrophages to kill *E. coli.*

**FIGURE 3:**
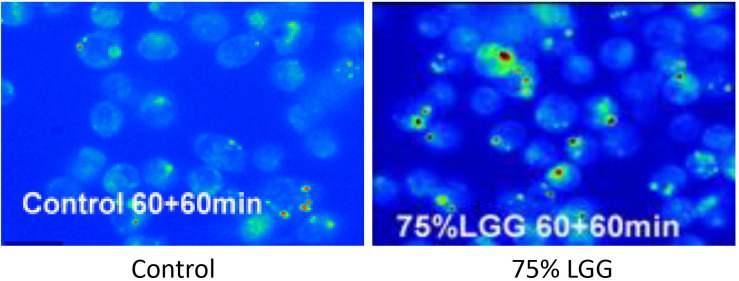
Killing of intracellular bacteria within J774 macrophages in the presence of LGG supernatant. Viable bacteria were observed following phagocytosis using acrdine orange and crystal violet staining assay. Microscopy images show control (**left**, no treatment), 75% LGG (**right** image) and are presented as pseudo-colored fluorescence images at 60 min time points during the killing phase period. Viable bacteria are shown in green and dead bacteria are shown in red.

To visualize the killed bacteria under the microscope, intracellular bacteria were stained with acridine orange/crystal violet to distinguish live from dead cells. The data shown in Figure 3, supported the killing curve data, and confirmed that macrophages treated with 75% LGG-CM exhibited a clear increase in bacterial killing compared to controls.

### Effect of NO and ROS Inhibitors on Bacterial Digestion

**FIGURE 4:**
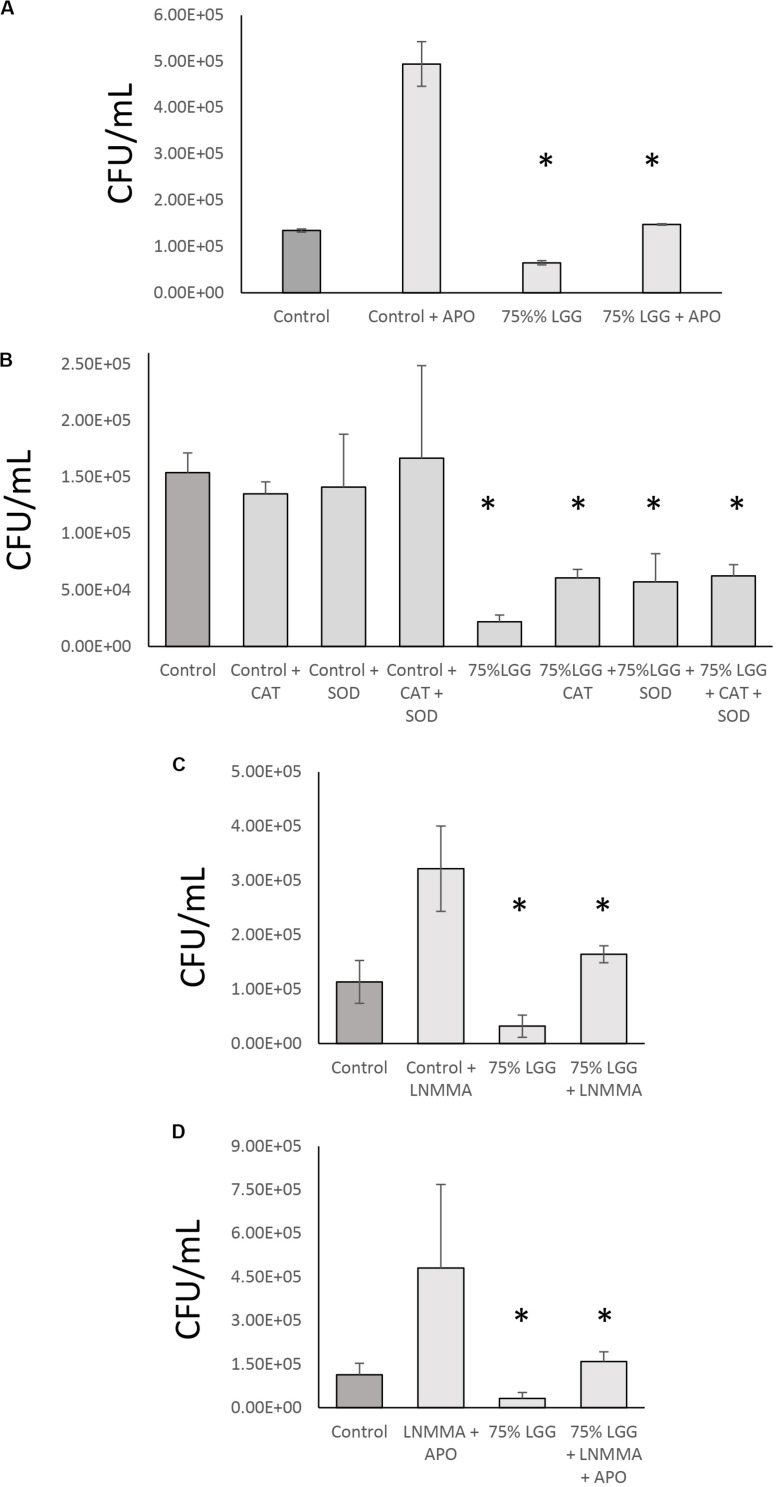
Role of NO and ROS inhibitor combination in bacterial digestion in the presence of LGG-CM. The effect of iNOS + NADPH oxidase inhibitors on bacterial digestion were investigated in the presence of LGG-CM. The inhibitors used were Apocyanin (Apo) = NADPH oxidase inhibitor **(A)**; Cell permeable variants of SOD and Catalase that scavenge reactive oxygen species **(B)**. LNMMA = iNOS inhibitor **(C)**. A combination of LNMMA and Apocyanin were used in **(D)**. Experiments were performed in duplicate and repeated three times. Error bars indicate ± standard error of the mean. Statistical analysis was carried out using Graph Pad Prism 3. The Dunnett’s multiple comparison post-hoc tests was performed after observing a significant difference from One way ANOVA. An asterisk (*) indicates significant difference (*p* < 0.01) between the treatments and control.

To identify the mechanisms involved in LGG-CM-stimulated macrophage bacterial killing, macrophages were incubated with inhibitors that are known to perturb different aspects of phagocytosis including L-NMMA, an inhibitor of the mammalian iNOS enzyme which catalyze the production of NO. In addition, cell-permeable variants of superoxide dismutase and catalase were used to scavenge free radicals. Apocyanin (Apo) which specifically inhibits NADPH oxidase that converts oxygen to superoxide radicals was also examined. As expected, all inhibitors suppressed bacterial killing in treated cells (Figure 4). Apo treatment resulted in a significant reduction in bacterial killing (*p* < 0.001) in both control cells and treated cells. However, the Apo treatment of LGG-CM treated cells (Figure 4A) did not return to the level of bacterial killing seen with the controls, suggesting LGG-CM stimulation of phagocytosis was multifactorial. This premise was suggested using the other inhibitors which demonstrated a similar trend (Figures 4B–D). Taken together, the data collectively demonstrate that the stimulation of bacterial killing by LGG-CM involves different canonical phagocytic stages that are blocked by these compounds.

### Stimulation of NADPH Oxidase by LGG-Conditioned Medium

**FIGURE 5:**
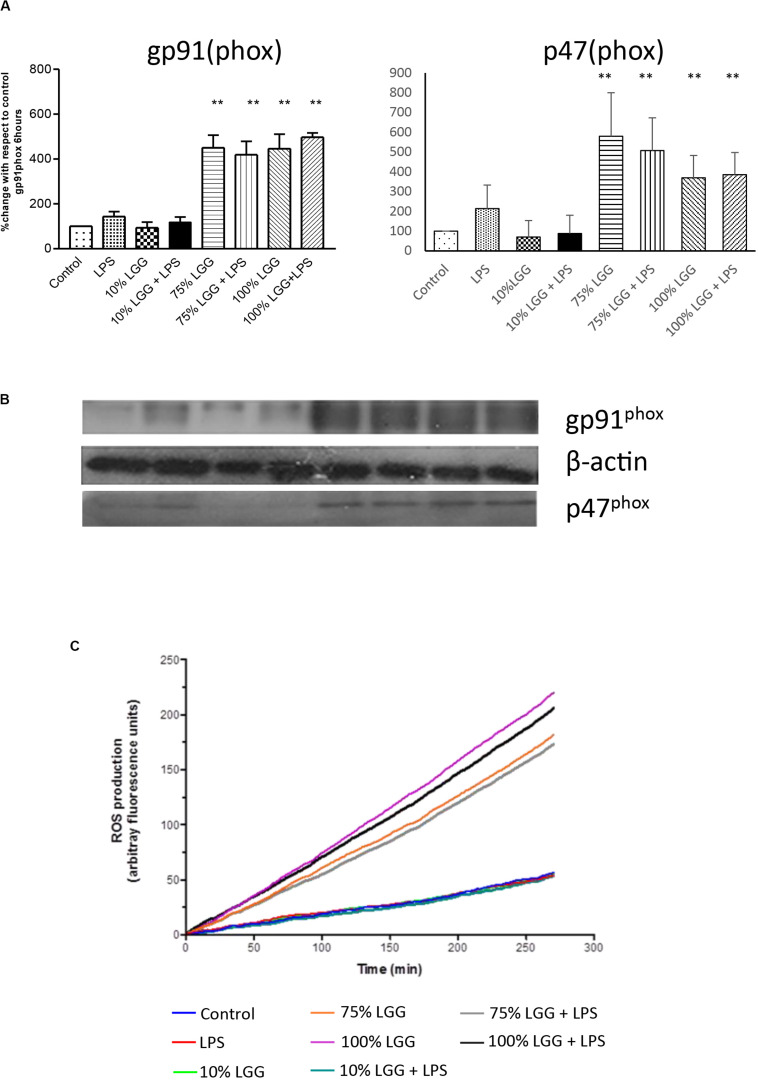
Effect of LGG-CM on reactive oxygen species induction. **(A)** Protein levels of the NADPH components p46(phox) and gp91(phox) were visualized and quantified by Western blot analysis as shown in **(B)**. Band quantitation of 3 independent experiments was performed by densitometry using Image J. Data shows relative protein levels with respect to the control; shown are means ± standard error of 3 independent experiments. A double asterisk (**) indicates a significant difference compared with the control (*p* < 0.01). **(C)** ROS production measured in real-time (between 0 and 280 min) by fluorescence using a ROS indictor dye DCFDA. The relative level of fluorescence is shown for each treatment given in the key.

To investigate the involvement of ROS in LGG-CM-stimulated macrophages further, Western blots were performed to visualise and quantify the relative expression of NADPH oxidase subunits gp91(phox) and p47(phox). Figure 5A shows densitometry data taken from the Western blots with a representative blot shown in Figure 5B. The data show untreated (control) levels of the protein significantly lower (*p* < 0.001) than LGG-CM-treated levels, consistent with an increased ability to kill bacteria, supporting our data. LPS and diluted samples of LGG-CM exhibited little effect on the expression of gp91 (phox) or P47 (phox). Taken together the Western blot data suggest an induction in NADPH expression, consistent with its role in bacterial killing.

### Visualization of ROS Production

To further assess the role of ROS induction in LGG-CM-treated cells, we measured ROS production in real time within J774 murine macrophages pre-loaded with the fluorescent dye 2,7-dichlorofluorescein diacetate (DCFDA; Figure 5C). Fluorescence was monitored every 2 min between 0 and 280 min to give a quantitative measure of intracellular ROS production. ROS production was stimulated when macrophages were exposed to the LGG-CM, with or without the addition of LPS (Figure 5C). LPS alone, or 10% LGG-CM did not affect ROS production within the macrophages. Our data support the Western blot and bacterial killing data that ROS production is stimulated in macrophages by exposure to the LGG conditioned medium (Figure 5C), suggesting secreted components by *Lactobacillus* have the ability to stimulate ROS via NADPH oxidase activity.

**FIGURE 6:**
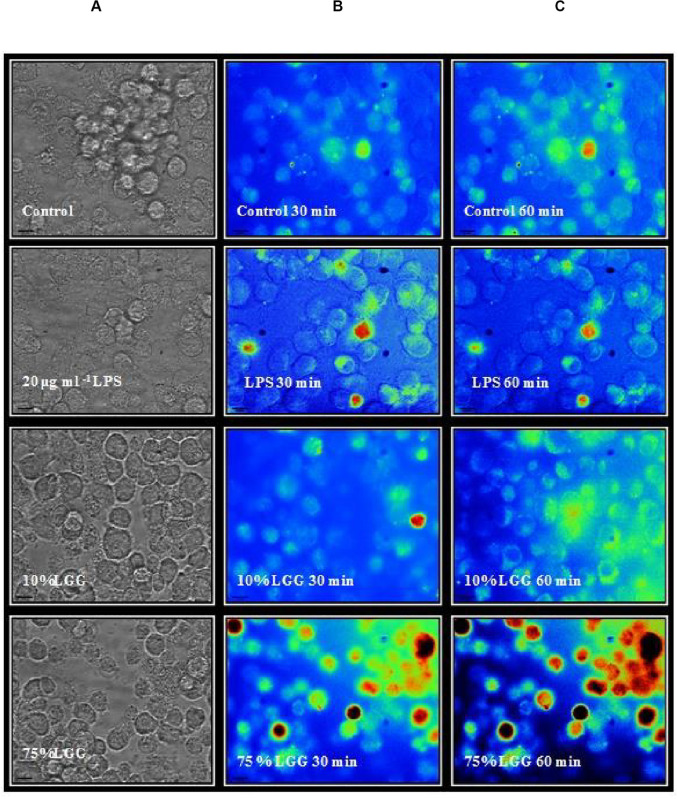
Fluorescence microscopy imaging of ROS production in macophages. The macrophages were labeled with H_2_DCFDA and treated with 20 μg ml^–1^ LPS, 10 % LGG-CM, and 75 % LGG-CM for 30 and 60 min. The images are representative of one of the three experiments performed. The vertical panels illustrate adjacently placed bright field **(A)** and pseudo colored fluorescence images **(B,C)**. Fluroescence corresponds to a heat map, blue = low, and red = high.

The DCFDA data were further supported by microscopy (Figure 6). ROS production from cells treated with LGG-CM macrophages were first loaded with H_2_DCFDA. Macrophages were exposed to different conditions including LGG-CM (10% LGG and 75% LGG) and 20 μg ml^–1^ LPS and cells were visualized by fluorescence microscopy as described in the Methods section. To demonstrate the fluorescent dye, images were psudocolored (Figure 6) with blue representing the coolest fluorescent spot, followed by shades of green, yellow, orange, and red for brighter intensity. It was evident from the images that macrophages treated with 75% LGG-CM demonstrated higher ROS production both at 30 min and 60 min compared to control, LPS treated or 10% LGG-CM treated macrophages.

### Nitric Oxide Production by LGG-CM

**FIGURE 7:**
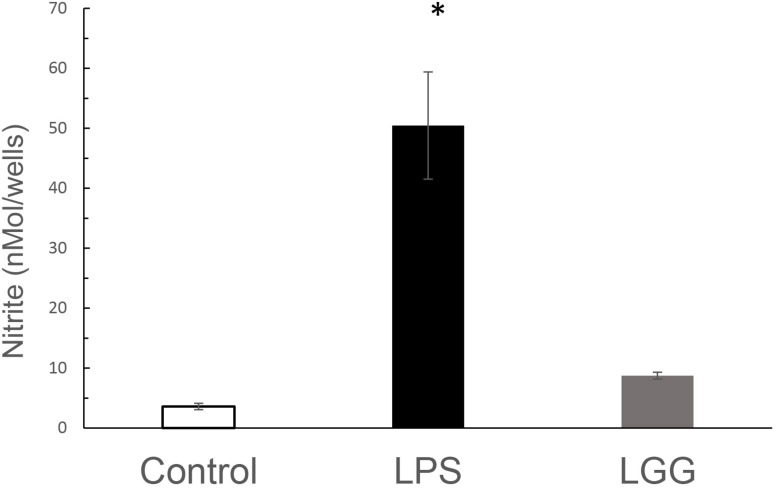
The effect of LGG-CM and LPS on NO production from J774 macrophages. Macrophages were treated with LGG-CM and LPS (1 μg ml^–1^). Accumulated nitrite was measured by Griess assay after 24 h. Results are the mean of 5 separate experiments performed in triplicate. Error bars indicate ± standard error of the mean. Statistical analysis was performed using one-way ANOVA followed by Dunnett multiple comparison test. Asterisk shows significance at *p* < 0.05.

To determine whether LGG-CM regulated NO production, J774 macrophages were treated with the LGG-CM for 24 h prior to determining nitrite levels. Nitrite is an indirect measure for NO production. LPS was used as a positive control. LGG-CM caused a small, observable but not significant increase in nitrite levels above control at following 24 h of incubation. LPS on the other hand induced significant elevation in NO production at 24 h (*p* < 0.001; Figure 7).

Taken together these experiments demonstrate that while LGG-CM-induced bacterial killing is multifactorial, involving multiple phagocytic pathways, it is strongly linked with the production of ROS through NADPH oxidase, with little significant input for NO production.

## Discussion

This study investigated the bacterial killing ability of the murine macrophage J774 cell line following exposure to different concentrations of cell-free conditioned medium from the probiotic bacterium *L. rhamnosus* GG (named LGG-CM). Cell free LGG-CM was used since it was shown to have immunomodulatory effect similar to live bacteria (Yan and Polk, 2012). This was also done to avoid interference of potential ingestion of the *Lactobacillus* bacteria by the macrophages. In this study, macrophages and *E. coli* were incubated for 60 min at a ratio of 1:50 which has been found to be essential to generate reproducible data in our study. The intracellular killing process was monitored for up to 280 min post incubation period, since phagocytic death of ingested bacteria takes approximately 2 to 4 h post ingestion (Levitz et al., 1986; Michaliszyn et al., 1995; Hacker et al., 2002; Philippe et al., 2003; Kirschnek et al., 2005). The rate of decrease in the total number of bacteria recovered during the post-incubation bacterial digestion period from the macrophages was used as a measure of the rate of intracellular killing.

Bacterial killing in macrophage occurs in the phagosomes. Fundamental changes in the acute chemical composition of phagosomes, and their subsequent fusion with endosomes, and lysosomes creates an environment, which becomes a highly acidic, oxidative and degradative milieu for killing of bacteria. During phagosome maturation, the phagosomes develop into a phagolysosome within which a range of anti-bacterial compounds such as antimicrobial peptides and reactive free radicals including ROS and NO are generated (Flannagan et al., 2009). These biochemical processes may take from several minutes to few hours depending upon the bacterial strain and the type of phagocytes such as neutrophils or macrophages (Nusse, 2011).

Regarding the experimental design of our study, there are several points that should be considered. The killing of bacteria in steadily maturing phagolysosomes could have an impact on viable bacterial cell recovery. In our experiments, the number of *E. coli* recovered from the control and LGG-CM-treated macrophages at 60 min was identical to the number of *E. coli* recovered at 30 min time points for both control and LGG-CM treatments, suggesting a limited bacterial killing occurs within the first 1 h of incubation period. Similar results were reported previously (Vincenti, 2010). Thus, in all experiments in this study, phagocytosis assays were performed following a 60-min incubation period. While our experiments also used a non-pathogenic species of *E. coli*, we accept that the role of phagocytosis against non-pathogens may be different as it would involve pathogenic strategies that we have not tested in the present study. Another caveat of this study is that the effect of LGG-CM on the killing of *Lactobacillus* bacteria itself is not studied. We envisage that components secreted by the *Lactobacillus* would be acting *in-trans*, with little access of the macrophage to the probiotic strain. We should also acknowledge that *E. coli*, like other species of bacteria, have ROS or NO evasion and defensive strategies that may impact the findings of this study. Thus, while our data report on bacterial killing, the defensive strategy of the bacterial species being tested has not been considered and may impact the elucidation of the mechanism involved. Finally, in this study we have used chemical inhibitors to provide information of the pathways that might be involved in LGG-CM stimulation of bacterial killing. However, we accept that inhibitors may have a variety of off-target effects and for this reason, we supported the inhibitor studies with Western blot analysis and in-cell monitoring of ROS.

Certain probiotic supernatants have previously been shown to have antibacterial properties on a number of bacterial species. This effect was suggested to be due to low pH of the supernatant (Tejero-Sariñena et al., 2012). This effect was found to be negated by neutralizing the pH. Additionally, in our study, probiotic conditioned media from *L. rhamnosus* had no direct antibacterial effect on the *E. coli* species we used in the study for up to 24 h. In contrast, when incubated with J774 macrophages, LGG-CM enhanced the rate of bacterial killing caused by macrophages. This was very much dependent on the concentration of LGG-CM used with 75% or 100% of the conditioned medium causing six times more *E. coli* killing. The increased rate of *E. coli* killing was found to be associated with the enhanced ROS production induced by LGG-CM in J774 macrophages.

The role of ROS in bacterial killing has previously been emphasized by the finding that it can attack both extra-cytoplasmic or cytoplasmic targets (in particular iron-sulfur cluster- containing proteins and DNA; Craig and Slauch, 2009). The increase in ROS production in response to LGG-CM in our studies was associated with the activation of NADPH oxidase as shown by Western Blot. In phagocytes, NADPH oxidase dependant ROS production is known to be crucial for microbial killing; however, excessive ROS production induces tissue injury and prolonged inflammatory reactions that contribute to inflammatory diseases. Thus, NADPH oxidase activation must be tightly regulated to limit ROS production.

Phosphorylation of p47^*phox*^ is essential for NADPH oxidase activation in phagocytes such as neutrophils (Li and Shah, 2004) and unphosphorylated p47^*phox*^ has been shown to have inhibitory effects on NADPH oxidase activity (Ray and Shah, 2005). Phosphorylation of p47^*phox*^ has been shown to relieve these inhibitory effects and at the same time increased NADPH oxidase activity by binding to membrane bound gp91^*phox*^ (Belambri et al., 2018). In the present study, NADPH oxidase subunits gp91^*phox*^ and p47^*phox*^ were measured at different time points. We did not observe any significant changes in the gp91^*phox*^ and p47^*phox*^ expression in the first 2 h of incubation with LGG-CM treatments (data not shown); whereas at 6 h of incubation, LGG-CM treated macrophages showed a significant increase in the expression of gp91^*phox*^ and p47^*phox*^ compared to untreated macrophages. This is in line with the findings from previous studies that NADPH oxidase activation occurs over the short term through phosphorylation of the enzyme subunits and over the long term by increased subunit expression (Li and Shah, 2004).

Recent findings demonstrated that LGG-CM is capable of stimulating a differential rate of ROS production from macrophages during the ingestion period and the digestion period. When macrophages were treated with a higher concentration of LGG-CM, the ROS production rate during the first hour of treatment was approximately 2 times higher than the ROS production rate during the next 280 min. LGG-CM mediated ROS production in the macrophages appears to be oscillatory (Nanjundaiah et al., 2016). This sort of ROS regulation has been previously demonstrated in plant root hairs and guard cells and also during pollen stigma interactions (McInnis et al., 2006; Monshausen et al., 2007; Takeda et al., 2008; Jammes et al., 2009). Detection of the oscillating pattern of ROS signals in plants clearly supports our current findings where our raw data for free radical production demonstrates an oscillatory pattern (not shown). This oscillatory pattern of ROS production in macrophage to LGG-CM may play a role in maintaining a nontoxic steady-state level of ROS, while allowing for the transient accumulation of ROS in particular subcellular locations. This pattern of ROS production may have a physiological significance and could act as signals (Mittler et al., 2004). In this study, the LGG-CM may activate macrophages to kill microbes through the production of ROS and perhaps through modulation of NO. This phenomenon might have physiological importance in shifting its immune function from degrading debris from tissues to killing invading microbes to present antigen to *T* cells.

In this study, although LPS has been shown to produce substantial levels of NO, it was found to have little effect on the bacterial killing process. Such data is inconsistent with some studies but clearly supported by others (Michlewska et al., 2009; Feng et al., 2011; Kapellos et al., 2016), suggesting different experimental design may be responsible for these different outcomes. On the other hand, LGG-CM has increased bacterial killing which was around three times faster than the LPS treatment despite inducing little NO when compared to LPS. It is worth noting, however, that NO has been shown to play an essential role in the anti-microbial and tumouricidal activity of murine macrophages (Netea-Maier et al., 2018; Ramachandran et al., 2018). NO, formed from arginine by nitric oxide synthases (iNOS), and its downstream reactive nitrogen intermediates (RNIs) are toxic to microbes and host cells via cysteine S-nitrosation of proteins, deamination of nucleic acids, and desaturation of lipids (Nathan and Cunningham-Bussel, 2013). There is evidence that reduction NO does not interfere with macrophage phagocytosis (Miyashita et al., 2018). This is inconsistent with our study using pharmacological inhibition of NO production with L-NMMA which was found to reduce bacterial killing, although, increase production of NO with LPS had no effect on bacterial killing mechanism. In our study probiotic conditioned medium seems to skew the balance of ROS and NO production via a complex and poorly understood mechanism.

In conclusion, we found that the bacterium LGG releases components during cell culture which enhances bacterial killing by macrophages, associated with increased generation of ROS via activation of NADPH oxidase. Small increases in NO may also have a role in the bacterial killing mechanism, however, it is probably a beneficial mechanism for probiotic bacterial to protect the host as excessive generation of NO can cause tissue damage. This is first report that shows the differential regulation of ROS and NO by probiotic bacteria on bacterial killing mechanism(s) by macrophages and this may have clinical importance in maintaining intestinal homeostasis.

## Data Availability Statement

The raw data supporting the conclusions of this article will be made available by the authors, without undue reservation.

## Author Contributions

YN performed the experiments and analyzed the data. MS designed the study, analyzed the data, and drafted the article. DW designed the study. ZA and AB reviewed the article. ZK performed the study design and reviewed the article. All authors contributed to the article and approved the submitted version.

## Conflict of Interest

The authors declare that the research was conducted in the absence of any commercial or financial relationships that could be construed as a potential conflict of interest.
